# Estimation of Health State Utility Values in Fabry Disease Using Vignette Development and Valuation

**DOI:** 10.36469/001c.71344

**Published:** 2023-04-10

**Authors:** Derralynn Hughes, Andrew Lenny, Koonal Shah, Louise Longworth, Giovanna Devercelli, Olulade Ayodele

**Affiliations:** 1 Royal Free London NHS Foundation Trust, University College London, United Kingdom; 2 PHMR, London, United Kingdom*; 3 PHMR, London, United Kingdom; 4 Takeda Development Center Americas, Lexington, Massachusetts, USA

**Keywords:** economic models, enzyme replacement therapy, Fabry disease, health-related quality of life, health state utilities

## Abstract

**Background:** Health state utilities are measures of health-related quality of life that reflect the value placed on improvements in patients’ health status and are necessary for estimation of quality-adjusted life-years. Health state utility data on Fabry disease (FD) are limited. In this study we used vignette (scenario) construction and valuation to develop health state utilities.

**Objectives:** The aim of this study was to use vignette construction and valuation to estimate health state utility values suitable for inclusion in economic models of FD treatments.

**Methods:** Health state vignettes were developed from semistructured qualitative telephone interviews with patients with FD and informed by published literature and input from an expert. Each vignette was valued in an online survey by members of the United Kingdom (UK) general population using the composite time trade-off (TTO) method, which aims to determine the time the respondent would trade to live in full health compared with each impaired health state.

**Results:** Eight adults (50% women) with FD from the UK were interviewed. They were recruited via various approaches, including patient organizations and social media. The interviewees’ responses, evidence from published literature, and input from a clinical expert informed the development of 6 health state vignettes (pain, moderate clinically evident FD [CEFD], severe CEFD, end-stage renal disease [ESRD], stroke, and cardiovascular disease [CVD]) and 3 combined health states (severe CEFD + ESRD, severe CEFD + CVD, and severe CEFD + stroke). A vignette valuation survey was administered to 1222 participants from the UK general population who were members of an external surveying organization and agreed to participate in this study; 1175 surveys were successfully completed and included in the analysis. Responses to TTO questions were converted into utility values for each health state. Pain was the highest valued health state (0.465), and severe CEFD + ESRD was the lowest (0.033).

**Discussion:** Overall, mean utility values declined as the severity of the vignettes increased, indicating that respondents were more willing to trade life-years to avoid a severe health state.

**Conclusions:** Health state vignettes reflect the effects of FD on all major health-related quality-of-life domains and may help to support economic modeling for treatment of FD.

## BACKGROUND

Fabry disease (FD), a rare, X-linked, inherited metabolic disease, is caused by insufficient activity of the lysosomal enzyme α-galactosidase A. This leads to cellular accumulation of globotriaosylceramide and its derivatives (eg, globotriaosylsphingosine), resulting in multisystemic organ manifestations that can include severe renal, cardiac, pulmonary, and neurological involvement.^1^ The prevalence of FD ranges from 1 in 17 000 to 1 in 117 000 in white male populations.^2^ In the United Kingdom (UK) general population, prevalence has been reported as 0.27 and 0.29 per 100 000 for male and female patients, respectively.^3^ FD is divided into classical and nonclassical phenotypes.^4^ Classical FD is characterized by a multisystemic organ manifestation; signs and symptoms include neuropathic pain, cornea verticillata, and angiokeratoma with early onset. The prevalence of classical FD ranges from approximately 1 in 22 000 to 1 in 40 000 in male patients.^2^ Nonclassical FD is characterized by a more variable, later-onset disease course than in classical FD, with patients experiencing a less severe disease that may affect only a single organ system, with cardiac involvement being the most common. The prevalence of nonclassical FD ranges from 1 in 1000 to 1 in 3000 and 1 in 6000 to 1 in 40 000 for male and female patients, respectively.^2^

Early treatment initiation with enzyme replacement therapy (ERT) is key to the treatment of affected major organs, including the prevention of increase in cardiac mass and stabilization of kidney function. Furthermore, early ERT initiation improves neuropathic pain, sweating, gastrointestinal symptoms, and reported hearing loss.^5–8^ Currently, 3 treatment options have been approved by the European Medicines Agency for the treatment of FD in the European Union, and these fall into 2 therapeutic classes: the first class of therapy is ERT and includes agalsidase alfa (Replagal, Takeda)^9^ and agalsidase beta (Fabrazyme, Sanofi Genzyme),^10^ both approved in 2001; the second class is chaperone therapy, for which migalastat (Galafold, Amicus Therapeutics; approved in 2016) is currently the only approved treatment.^11^

Agalsidase alfa is indicated for the long-term treatment of patients with a confirmed diagnosis of FD. Pivotal clinical studies in FD, as well as studies in real-world settings, have shown that treatment of FD with agalsidase alfa leads to measurable and sustained clinical benefits.^1,7,8,12,13^ However, data on the health state utility values of patients with FD are limited,^14–16^ and studies investigating the effect of ERT on health-related quality of life (HRQoL) have been inconclusive.^17^

A health state utility value is a measure of preference or value for a given state of health. The values of health state utilities are necessary for the estimation of quality-adjusted life-years, which is the widely used metric for cost-effectiveness analyses^18^; however, generating data for health state utilities in rare diseases is often challenging. Utility data generation studies, which collect data using preference-based measures of HRQoL or mapping studies, require large sample sizes and so are not feasible for FD owing to its rarity.^19^ A vignette, sometimes called a “scenario,” “health state vignette,” or “health state,” is a description of the impact of a medical condition that can be valued in a preference elicitation task to obtain a utility estimate.^20^ To generate the necessary health state utility data, a vignette is constructed to describe each of the frequently occurring states associated with a condition and its treatment, in a form that can be valued by survey respondents using standard valuation methods. The vignettes are usually based on findings from interviews with patients, caregivers, and/or healthcare professionals. They can incorporate a range of information about the impact of the condition and its treatment.^19^

This study aimed to use vignette construction and valuation to estimate health state utility values for FD that could then be used in cost-utility analyses to inform healthcare decisions in patients with FD.

## METHODS

### Vignette Development

Adults (aged ≥18 years) who were living in the UK and had a diagnosis of FD were recruited, in collaboration with the healthcare research agency Global Perspectives (Reading, UK), to understand patients’ lived experience of FD and to generate the descriptions for health state vignettes. Global Perspectives is a provider of data for clinical noninterventional studies. They used a mix of recruitment approaches, including tailored interviews, online surveys, and mobile apps. Individuals with FD interested in participating in the research contacted the recruiter, and a screening telephone call was then conducted to check eligibility for the study. If eligible, the participant was sent an information sheet and consent form to take part in the study. Health state vignettes describing the impact of FD on patients’ HRQoL were developed from semistructured qualitative telephone interviews with the recruited patients to gain their perspective on the impact of this disease. Patient interviews (or qualitative interviews) were performed to develop vignettes describing specific complications for FD; therefore, a full thematic analysis was not conducted, but responses were analyzed drawing on thematic analysis techniques.

A discussion guide was created to carry out the patient interviews. The content of this guide was informed by a focused review of the literature concerning the impact of FD on patients’ quality of life.^1,12,13,21^ The discussion guide included semistructured questions about the nature, terminology, frequency, severity, and impact of known physical and neurological symptoms, adverse events, and complications. There were also questions about any other important symptoms, adverse events, or complications that had not already been discussed.

The findings from the patient interviews informed the development of a health state vignette primarily focused on pain-related symptoms, and 2 health state vignettes describing moderate and severe clinically evident FD (CEFD). However, further health state vignettes were required for the main complications associated with progression of this disease, namely cardiovascular disease (CVD), end-stage renal disease (ESRD), and stroke. Most of the patients interviewed were in the early stages of FD progression and had limited or no experience of the 3 complications of interest (4 patients reported CVD [of whom 2 patients reported arrhythmia], 2 patients reported ESRD, and none reported stroke). Therefore, information to develop these health state vignettes was sought from external sources. For the CVD and stroke health states, the descriptions developed by Matza et al^22^ were used. The process used by Matza et al to develop these states was considered methodically rigorous, with multiple rounds of review with clinicians and further refinement via a pilot study with a sample of the UK general population (n = 200).^22^ To develop the ESRD vignette, descriptions of the effects were drawn from the data collected in the qualitative interviews and from online sources, such as the websites of the UK National Health Service (NHS) and the National Kidney Foundation.^23,24^ Health state vignettes were refined using an iterative process and incorporated feedback from an independent clinical expert in FD, to ensure that they were clinically accurate and reflected real health states associated with FD. Health state vignettes based on combinations of disease complications were planned but were not included in the final valuation survey owing to their complexity; instead, these were calculated after data collection.

### Vignette Valuation

The developed health state vignettes were reviewed and the utility value for the health state for each vignette was determined by members of the UK general population using the composite time trade-off (TTO) method.^25^ This method combines conventional TTO and lead-time TTO approaches. It starts with the conventional TTO for all health states: if the respondent considers the health state to be “better than dead,” the “better than dead” task of the conventional TTO is used. However, if the respondent considers the health state to be “worse than dead,” the lead-time TTO is used. The goal is to determine how many years of life in full health the respondent considers equivalent to a fixed number of years in each impaired health state. Respondents were presented with a series of choice tasks, each involving 2 hypothetical lives. They were asked to choose between living a longer life in the impaired health state or a shorter life in full health. Depending on which life path was chosen, the amount of time in full health was altered until a point of preferential indifference was reached, at which point the respondent’s utility value for the health state could be calculated. Utility values were anchored on a scale with 0 representing dead and 1 representing full health. If a respondent considered a health state to be “worse than dead,” a negative utility value was assigned to that health state.

The implementation of the TTO in this study followed the EQ-5D-5L valuation protocol as far as feasible.^25^ The use of the composite TTO approach, description of the health state anchors, and the iteration procedures followed the published protocol. An online self-completion survey was administered to 1222 participants from the UK general population between June 18 and July 7, 2021, to implement the TTO tasks. The survey was administered to a sample of members of an external surveying organization (Qualtrics, Provo, Utah, USA). Respondents agreed to be invited to participate in this study. Quota sampling was applied to ensure a representative sample from the UK in terms of age and gender. The sample size was informed by the EQ-5D-5L valuation protocol for TTO valuation studies, which recommends a sample of at least 1000 respondents for EQ-5D-5L valuation studies.^26^ Respondents were presented with a 6-minute instructional video introducing the TTO method and explaining how to complete TTO tasks. Given that the survey was conducted online without interviewer assistance, the wording and formatting of some of the instructions, including the inclusion of an instructional video, differed for clarity in an online setting. Respondents then completed a practice TTO task, valuing a mild generic health state unrelated to FD. The main valuation began after this, in which each respondent completed 9 further TTO tasks—1 task for each of the 6 health state vignettes, and 1 for each of the 3 combination health state vignettes.

### Statistical Analysis

Respondent background characteristics were reported using counts and proportions, and compared with the most recent UK census data to assess representation across the sample.^27^ The general health of the respondents was compared with the data collected from the 2009 Adult Dental Health Survey (ADHS).^28^ Mean utility values were calculated for individual health state vignettes in accordance with EuroQol protocols.^29^ For the combination vignettes, the additive, multiplicative, and minimum methods were considered. To evaluate which approach yielded the most accurate data, the utilities for severe CEFD and each complication health states were combined using the additive and multiplicative approaches. These were compared with the utility data generated by the survey for the health states combining complications with severe CEFD. The multiplicative approach was chosen as the preferred method because it yielded values closest to the observed values generated by the online valuation survey.

Subgroup analyses were conducted to examine potential differences in preferences across different background characteristics of the sample. A linear regression model was conducted for the analysis of each health state separately, with utility value as the dependent variable. The background characteristics and survey completion time were included as explanatory variables. Sensitivity analyses were conducted with varying exclusion criteria applied, based on the total number of logical inconsistencies, to assess the impact of exclusion. This study used exclusion criteria similar to those in published national valuation studies.^30^ Responses were checked for logical inconsistencies, with inconsistency being defined as the valuation for “health state A” exceeding the valuation of “health state B” when “health state B” represented logically better quality of life than “health state A” (ie, when “health state B” was at least as good as “health state A” on all aspects described in the vignette). The mean values were expected to follow a logical pattern from least to most severe for the health states for which this difference in severity was discernible (pain → moderate CEFD → severe CEFD) and (ESRD → severe CEFD + ESRD, CVD → severe CEFD + CVD, stroke → severe CEFD + stroke).

Another sensitivity analysis examined the impact of excluding respondents who completed the survey unusually quickly (based on a prespecified criterion of completing the survey in <50% of the median survey time). A scenario analysis was conducted to assess the impact of removing respondents with a major inconsistency (eg, valuing the pain health state—the mildest of the health states—as worse than any other health state). The impact of excluding respondents who gave the same value for all health states, thereby failing to distinguish between states of differing severity, was also assessed. This included “nontraders”—respondents who were not willing to trade any time in any of the TTO tasks—who were assigned a value of 1 to all health states.

## RESULTS

### Vignette Development

Eight adults with FD living in the UK were recruited and interviewed for 45 to 60 minutes in collaboration with the healthcare research agency Global Perspectives. Of these respondents, 4 (50%) were women and 5 (62.5%) were receiving FD-specific treatment. Based on age at symptom onset, 2 respondents were reported to have early-onset FD and 6 respondents late-onset FD. Phenotype information was not collected for these participants. Three patients (37.5%) were aged 30 to 39 years, 3 (37.5%) were aged 40 to 49 years, 1 (12.5%) was aged 50 years or older; the age of 1 patient was not disclosed. The most prevalent signs and symptoms mentioned were fatigue (n = 8), acroparesthesia (n = 8), gastrointestinal problems (n = 7), angiokeratoma (n = 7), and anhidrosis (n = 6) (Table 1). Sweating problems were experienced by all 8 patients, 6 of whom experienced anhidrosis and 2 of whom experienced hyperhidrosis. It was decided to include anhidrosis and to exclude hyperhidrosis from the pain and CEFD vignettes because anhidrosis was more prevalent and, based on the responses provided, seemed more important to daily activities. Four patients mentioned that they had experienced depression in some form during their lifetime, which is a symptom that was not included in the vignettes. Depression was usually attributed to the current and future negative health outcomes associated with the patient’s disease or with the prospect of passing FD on to their children. Another symptom that was not included in the vignettes was tinnitus, which was reported by 5 patients; however, it was excluded because none of these patients described it as having a substantial impact on their quality of life or as impeding their daily activities in any way.

**Table 1.** Signs and Symptoms Described During Patient Interviews

**FD-related Symptoms**	**Symptom Description**	**Instances Mentioned**
Fatigue	Having a moderate to severe impact on patients’ HRQoL and daily activities, including their ability to get out of bed in the morning, to do their job properly, and to interact with their families. The frequency of these bouts of fatigue varied from 3-4 times per month to at least once per day	8
Acroparesthesia	A painful sensation ranging from a mild hot or tingling sensation to a severely painful burning sensation. This pain was usually experienced in the hands and/or feet and worsened in hot weather. When severe, it can be very debilitating, affecting patients’ ability to perform day-to-day activities. At its mildest, pain was experienced infrequently (at least once per week); at its worst, it caused pain daily	8
Gastrointestinal problems	Stomach cramps and diarrhea that severely limited patients’ daily activities and their ability to plan ahead	7
Angiokeratoma	Red/dark spots covering various areas of the body including the lower stomach, legs, and back. Highly embarrassing and negatively affecting self-confidence and social activities	7
Anhidrosis (lack of sweating)	Patients who experienced this symptom indicated that it was constantly an issue for them, and it was exacerbated by hot weather or exercise	6

Abbreviations: FD, Fabry disease; HRQoL, health-related quality of life.

**Figure 1.**
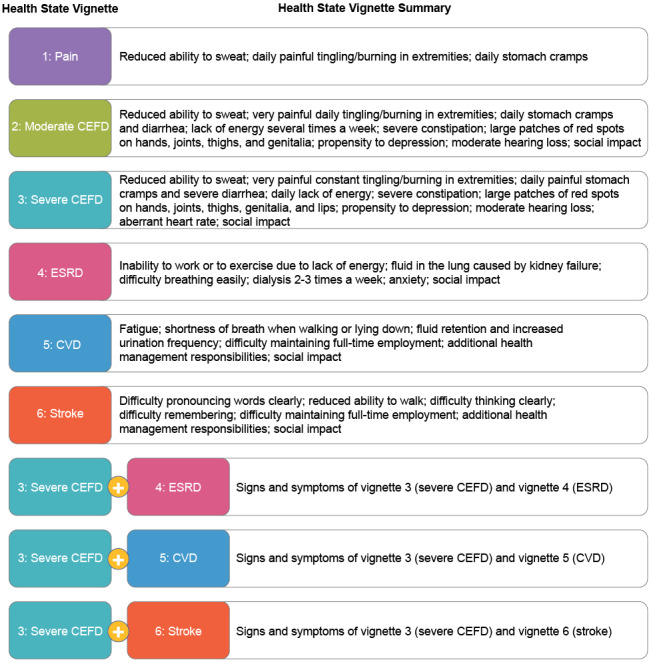
Summary of Health State Vignettes Abbreviations: CEFD, clinically evident Fabry disease; CVD, cardiovascular disease; ESRD, end-stage renal disease.

The interviewees’ responses, evidence from published literature, and input from a clinical expert informed the development of 6 health state vignettes covering the following: pain, moderate CEFD, severe CEFD, ESRD, stroke, and CVD. In addition, 3 combined health state vignettes were developed, covering severe CEFD + ESRD, severe CEFD + CVD, and severe CEFD + stroke (Figure 1).

### Vignette Valuation

Of the 1222 participants from the UK general population who accessed the survey, responses to TTO questions were received from 1175 adults (Table 2). Forty-seven respondents were automatically excluded because they completed the survey in less than 10.5 minutes (more quickly than 50% of the median completion time), based on prior agreement with the panel provider and informal piloting of the survey. Completing the survey this quickly was deemed implausible if the respondent had watched the explanatory video and read the instructions in full. The median (range) completion time was 21.0 (8.23-489.55) minutes. The recruited sample was representative of the UK general population in terms of age and gender according to the 2011 UK Census,^27^ with the exception of those aged 75 years or older, who were underrepresented. Differences between the study sample and UK census and ADHS were analyzed using a χ^2^ test (age, *P* = .784; current health, *P* = .007; gender, *P* = .848). The data collected here were similar to the data collected in the ADHS in terms of the proportions of the respondents choosing “very bad” and “bad” in the current health categories. The proportion of respondents reporting “very good” in the current health categories (14.6%) was approximately half of that reported in the ADHS (35.8%), and the opposite was observed for the proportion of respondents reporting “fair” health (30.0% vs 15.7%). There was also a difference in the proportion of respondents reporting “good” health compared with ADHS data, although this difference was smaller (7.5% vs 43.1%).

**Table 2.** Valuation Survey: Respondent Demographics

**Characteristic**	**Respondent Sample, n (%)^a^**	**Proportion of UK Census 2011, %**
Gender		
Male	605 (51.5)	48.6
Female	563 (47.9)	51.4
Other	4 (0.3)	NA
Prefer not to say	3 (0.3)	NA
Age, years		
18-24	129 (11.0)	11.9
25-34	214 (18.2)	17.1
35-44	211 (18.0)	17.8
45-54	231 (19.7)	17.5
55-64	207 (17.6)	14.9
65-74	148 (12.6)	8.7
≥75	35 (3.0)	7.8
Education level		
Primary school	5 (0.4)	NA
Secondary school, up to age 16 years	242 (20.6)	NA
Higher or secondary or further education	321 (27.3)	NA
College or university	458 (39.0)	NA
Postgraduate degree	142 (12.1)	NA
Prefer not to say	7 (0.6)	NA
Current health	Self-reported^b^	ADHS 2009
Very good	172 (14.6)	35.8
Good	558 (47.5)	43.1
Fair	353 (30.0)	15.7
Bad	73 (6.2)	4.3
Very bad	20 (1.7)	1.1

Abbreviations: ADHS, Adult Dental Health Survey; NA, not applicable.

^a^n = 1175.

^b^The self-reported current health of the sample was compared with the current health data collected in the ADHS in 2009.

### Health State Utility Values

Pain was valued the highest at 0.465, and severe CEFD + ESRD was valued the lowest at 0.033 (**Table 3**). For the main complications of FD, the lowest mean utility value was for the ESRD health state, followed by the CVD health state and the stroke health state (0.119, 0.278, and 0.385, respectively). Standard deviation values ranged from 0.560 to 0.675 and grew broader as health state severity increased (**Table 3**). No health state had a negative health utility (ie, none was considered “worse than dead” on average). Given that the patient interviews were completed ahead of the quantitative online study, it would not have been possible to explore directly the order of values for the health states in the patient interviews.

**Table 3.** Health State Utilities

**Health States Valued in the Survey ^a^**	**Mean (SD)**	**Median**	**Standard Error**	**95% Confidence Limits**
Pain	0.465 (0.560)	0.6	0.016	0.433 to 0.497
Moderate CEFD	0.203 (0.637)	0.425	0.019	0.166 to 0.240
Severe CEFD	0.156 (0.658)	0.350	0.019	0.119 to 0.193
ESRD	0.119 (0.670)	0.275	0.020	0.074 to 0.150
CVD	0.278 (0.630)	0.5	0.018	0.252 to 0.314
Stroke	0.385 (0.606)	0.5	0.018	0.350 to 0.420
Severe CEFD + ESRD	0.033 (0.672)	0.0	0.020	−0.006 to 0.071
Severe CEFD + CVD	0.081 (0.675)	0.0	0.020	0.042 to 0.119
Severe CEFD + stroke	0.111 (0.672)	0.2	0.020	0.073 to 0.150

Abbreviations: CEFD, clinically evident Fabry disease; CVD, cardiovascular disease; ESRD, end-stage renal disease.

^a^n=1175.

### Combination Health State Analyses

The utility values for the combination health states have been calculated using 3 combination methods and are shown in **Table 4**. Of the 3 methods used, the additive method was the only one to produce negative mean utility values, except for the CVD + stroke combination health state. To evaluate which approach yielded the most accurate data, utilities for severe CEFD and each complication health state were combined using the additive and multiplicative methods, and compared with the utility data generated by the survey. The minimum approach was excluded from this comparison owing to previously demonstrated bias in favor of younger cohorts.^31^ The multiplicative approach yielded values closest to the observed values generated by the online valuation survey when using pain—the least severe health state—as the baseline level of utility (**Table 5**). This suggests that, when assessing health states that include more than 1 complication, it is more accurate to use the values generated by the multiplicative method.

**Table 4.** Calculated Utility Values for Combination Health States

**Combination Health State**	**Additive Approach**	**Multiplicative Approach^a^**	**Minimum Approach**
(Severe CEFD + ESRD) + (severe CEFD + CVD)	−0.042	0.017	0.033
(Severe CEFD + ESRD) + (severe CEFD + stroke)	−0.012	0.024	0.033
(Severe CEFD + CVD) + (severe CEFD + stroke)	0.036	0.058	0.081
(Severe CEFD + ESRD) + (severe CEFD + CVD) + (severe CEFD + stroke)	−0.087	0.012	0.033

Abbreviations: CEFD, clinically evident Fabry disease; CVD, cardiovascular disease; ESRD, end-stage renal disease.

^a^Recommended values.

**Table 5.** Comparison of Utility Combination Methods

**Complication Health State**	**Mean Utility Value**	**Additive Approach (Pain as Base Health)**	**Multiplicative Approach (Pain as Base Health)**
Severe CEFD + ESRD	0.033	−0.19	0.040
Severe CEFD + CVD	0.081	−0.031	0.093
Severe CEFD + stroke	0.111	0.076	0.129

Abbreviations: CEFD, clinically evident Fabry disease; CVD, cardiovascular disease; ESRD, end-stage renal disease.

### Subgroup Analysis

The variables that were statistically significant (*P* < .05) in explaining variance in health state utility values were age, education level, and survey completion time (**Supplementary Table S1**). Survey completion time was statistically significant for explaining the variance in utility values for all health states and had a small negative impact on mean utility (ie, the longer respondents spent on the survey, the lower the utility scores were for every health state). Respondent age was statistically significant for explaining the variance in utility values for the stroke and severe CEFD + stroke health states. Although nonsignificant, there was a small, negative relationship between age and every health state, except for the pain health state, for which a small positive relationship was observed. Respondent education level had a marginally positive, statistically significant relationship with mean health state utilities for the moderate CEFD, severe CEFD, and CVD health states. Respondent gender and current health status were not statistically significant variables in explaining the variance in utility values for any of the health states.

### Sensitivity Analyses

TTO surveys typically produce responses that may be considered “inconsistent.” However, there is no consensus in the literature as to what constitutes an “inconsistent” response or when to exclude such responses. For this reason, sensitivity analyses were conducted using alternative exclusion criteria to assess the impact of exclusion on the results. In this study, the data features that were considered inconsistent, illogical, or otherwise problematic were instances in which respondents met any of the following criteria: completed the survey in less than 10.5 minutes (less than 50% of median completion time); valued all health states as the same; or did not value pain as the least severe health state. In each of the 7 scenarios tested using alternative rules for exclusion of data (**Supplementary Table S2**), the mean utility values followed the same logical pattern observed in the main analysis. “Worse than dead” values were observed in all but 2 scenarios tested, namely scenario 2 (in which respondents who gave the same value to each health state were excluded) and scenario 3 (in which respondents who did not value pain as the least severe health state were removed). The highest mean utility values across all health states were observed in scenario 3. The lowest mean utility values were observed in scenario 4, for which data from respondents who showed any inconsistencies (by meeting any of the 3 criteria listed above) were removed.

## DISCUSSION

We report the findings of an online vignette valuation survey that was designed to estimate the impact of FD on patients’ HRQoL. The vignettes were based on qualitative interviews performed with 8 patients with FD in the UK and then completed by 1175 adult participants from the UK general population, which were validated by a clinical expert to ensure clinical accuracy and reflection of health states associated with FD. Of the complication health states, the lowest mean utility value was observed for the ESRD health state, followed by the CVD health state, and then the stroke health state. The same pattern continued for the combination health states. Health state utilities are normally skewed with medians larger than means because of the impact of outlier responses, notably giving a value of −1 to a health state^32^; median values remained higher than mean values when observations from respondents who completed the survey in less than 10.5 minutes were included. Values were lowest for the severe CEFD + ESRD health state and highest for the pain health state. These findings highlight a potential difference between the impact of the early-onset, more severe, classical FD and the late-onset, less severe, nonclassical FD on patients’ HRQoL, because someone with nonclassical FD may experience few or none of the symptoms described in either the moderate or severe CEFD health state.

Compared with the utility values for similar health states in the literature, the mean utility values for each health state generated in this study were low, but comparison with EQ-5D health states of similar values suggest that these results are plausible. For example, the value set for England reported by Devlin et al,^33^ based on the EQ-5D-5L, found that the utility value for health state 23245 is 0.247 (11111, best health state; 55555, worst health state). Typically, other studies that were discussed sought utility values from patients, whereas our study sought utility values from the general public, consistent with recommendations from the National Institute for Health and Care Excellence.^34^ In a study by Rombach et al,^16^ a Markov model was developed to assess the cost-effectiveness of ERT for patients with FD. Mean utility values per year by disease state were estimated using the TTO method. These health states and associated utility values were no symptoms (0.87), acroparesthesia/symptomatic (0.76), single complication (0.74), and multiple complications (0.58).

The single complication health state shown in Rombach et al is considerably higher (0.74) than the utility values observed for the single complication health states generated in our study (0.119-0.385). There are some factors that could at least partly explain this difference. First, the patients in the Rombach et al study could have adapted to their condition and, hence, underestimated what it means to be in “full health.” This would bias their utility values upward and is a well-researched phenomenon.^35^ Second, those patients were all receiving ERT, which could have affected their utility values. Third, the health states in that study were described using EQ-5D, which fails to capture the impact of FD on certain dimensions of HRQoL. These include embarrassment, fatigue, and hearing loss,^36–40^ and FD affects all 3 of these dimensions of health to varying degrees.

In a study by Arends et al,^14^ the effect of sex, phenotype, age, different states of FD severity, pain, and ERT on EQ-5D utilities were evaluated. This study generated utility values for a wide range of health states, which were also higher than the values generated in our study. This disparity can also be partly explained by patient adaptation bias and the lack of validity of EQ-5D in relevant dimensions of health that are affected by FD. The results of this study also suggest that a very small proportion of patients reported extreme problems with pain and performing usual activities. This is at odds with the results of the qualitative interviews and the input from the clinical expert in our study. The values in that study were much higher than those described by Miners et al,^15^ who reported values similar to the results from our study (eg, a mean value of 0.56 for male patients with classical FD who had not been treated with ERT).

The utilities generated for the single complication health states were also compared with utility values of similar health states in the literature. Liem et al^41^ reviewed health state utilities generated using the TTO method for patients with ESRD undergoing hemodialysis and found utility values ranging from 0.39 to 0.89. The mean ESRD health state utility value generated in our study (0.119) lies below this range. A possible reason could be the inclusion of the description of how patients with FD and ESRD are unable to work owing to their extreme fatigue. The inclusion of very extreme levels of fatigue was requested by the clinical expert who reviewed the health state vignettes. If this level of fatigue is not matched by at least some of the health states reviewed by Liem et al, then this could explain why the utility values generated by the current study are comparatively low.

A systematic review of studies reporting utility values following a stroke identified that a wide range of values was reported.^42^ Mean reported values ranged from 0.30 to 0.90 for a minor stroke and from −0.08 to 0.71 for a major stroke. Similarly, a systematic review of EQ-5D utility values in CVD found that mean values ranged from 0.24 to 0.90.^43^ The CVD and stroke health states in our study were largely informed by the health states used by Matza et al^22^; however, the descriptions were shortened and some of the wording was amended to make the descriptions more appropriate for patients with FD. In Matza et al, the mean utility values for the chronic heart failure and stroke health states were 0.57 and 0.52, respectively, whereas the utility values observed for CVD and stroke health states in our study were 0.278 and 0.385, respectively.^22^ This is a substantial difference, and the order of severity of these health states is reversed compared with the mean utility values observed in our study. Although the reasons for this disparity cannot be identified, the change in wording and length of the descriptions, along with a different mode of administration used in Matza et al, could be causative factors.

This was a robust, iterative process that allowed for a high degree of confidence that the vignettes included in the valuation survey reflected the health states included in economic modeling and accurately represented the lived experiences of patients with FD. Most of the patients interviewed were not yet at the stage of disease to have experienced the 3 complications of interest, but this was mitigated by rigorous evidence from the literature.^22–24^ Although a systematic review of the literature on the effects of FD on HRQoL may have been informative in the development of vignettes, the most burdensome signs and symptoms appear to have been adequately captured by the methodology employed in this study; however, depression was excluded because it is challenging to capture the range of potential causes of depression within a health state vignette without making it considerably more complex. Pain, difficulties surrounding heritability, and a negative health perception have been identified among the potential causes of depression in FD, together with more subjective factors, such as uncertainty about the future and stigmatization.^44^ Furthermore, the valuation of health states that contain uncertainty can be methodologically challenging.

There are limitations to this research. The preferred approach of eliciting surveys using the TTO method is face-to-face interviews, but the COVID-19 pandemic dictated the implementation of a self-completed online valuation approach; however, there was no evidence to suggest that the online approach had a negative impact on data quality. Other limitations included the small number of patients with FD who contributed to the development of the health state vignettes and the fact that they were predominantly in an early stage of the disease course. Further research to quantify the difference in HRQoL impact between patients with classical and nonclassical FD and any differences between male and female patients, as well as including patients with advanced disease, would be informative.

## CONCLUSIONS

The findings of an online vignette valuation survey that was designed to estimate the impact of FD on the HRQoL of patients have been detailed. Health state vignettes included summaries of the effects of FD on all major HRQoL domains. Overall, mean health state utility values followed a declining pattern as the severity of the vignettes increased, implying that respondents were more willing to trade life-years to avoid a severe health state than to avoid a less severe health state. This research highlights the considerable health burden in patients with FD and adds to the limited body of evidence for health state utility values for this rare disease. These utility values can potentially be used in economic modeling—such as cost-utility analyses—to determine cost-effectiveness of different FD treatments.

### Author Contributions

D.H., A.L., K.S., L.L., G.D., and O.A. provided substantial contributions to the conception and design of this study and the interpretation of the data, were involved in drafting the work or revising it critically for important intellectual content, approved the final version to be published, and have agreed to be accountable for all aspects of the work in ensuring that questions related to the accuracy or integrity of any part of the work are appropriately investigated and resolved.

### Disclosures

D.H. reports consulting and speaker fees from Amicus Therapeutics, Chiesi, Freeline Therapeutics, Idorsia, Protalix BioTherapeutics, Sanofi Genzyme, and Takeda outside the submitted work. A.L., K.S., and L.L. are or were employees of PHMR, London, UK at the time the study took place. A.L. is a current employee of Decisive Consulting Ltd, London, UK. K.S. is a current employee of the National Institute for Health and Care Excellence, UK. G.D. and O.A. are employees of Takeda Development Center Americas, Inc. and stockholders of Takeda Pharmaceuticals Company Limited.

### Ethical Approval

The UK Health Research Authority decision tool indicated that approval from an NHS Research Ethics Committee was not required for this study. Ethics review and approval was provided by an independent reviewer, David Carpenter (NHS Research Ethics Committee chair), working under the auspices of the Association of Research Managers and Administrators, in March 2020. Patient consent for publication was not required because de-identified data were used for analyses in this study.

### Data Sharing

The data that support the findings of this study are available from the corresponding author upon reasonable request.

## Supplementary Material

Online Supplementary Material
