# Supplementary material for: Estimation of Health State Utility Values in Fabry Disease Using Vignette Development and Valuation
Source: J Health Econ Outcomes Res. 2023 Apr 10;10(1):80–8. doi: 10.36469/001c.71344 (PMC10095067; doi:10.36469/001c.71344)
Supplement: Online Supplementary Material [file jheor_2023_10_1_71344_155967.pdf]

Journal of Health Economics and Outcomes Research

Online Supplementary Material

Estimation of Health State Utility Values in Fabry Disease Using Vignette Development and Valuation. *JHEOR*. 2023;10(1):80-88. doi:10.36469/jheor.2023.71344

Table S1: Variance in Health State Utilities

Table S2: Sensitivity Analyses

This supplementary material has been provided by the authors to give readers additional information about their work.

This is an open-access article distributed under the terms of the Creative Commons Attribution 4.0 International License (CCBY-4.0). View this license's legal deed at http://creativecommons.org/licenses/by/4.0 and legal code at http://creativecommons.org/licenses/by/4.0/legalcode for more information.

Table S1. Variance in Health State Utilities

Health State	Independent Variables	*P* < .05	Coefficient of Variation
Pain	Age		0.014
	Education level		0.031
	Completion time	✓	0.000
Moderate CEFD	Age		–0.013
	Education level	✓	0.047[a]
	Completion time	✓	0.000
Severe CEFD	Age		–0.016
	Education level	✓	0.039[a]
	Completion time	✓	0.000
ESRD	Age		-0.022
	Education level		0.036
	Completion time	✓	0.000
CVD	Age		–0.013
	Education level	✓	0.039[a]
	Completion time	✓	0.000
Stroke	Age	✓	–0.038[a]
	Education level		0.008
	Completion time	✓	0.001
Severe CEFD + ESRD	Age		–0.008
	Education level		0.029
	Completion time	✓	–0.105
Severe CEFD + CVD	Age		–0.014
	Education level		0.027
	Completion time	✓	–0.092
Severe CEFD + stroke	Age	✓	–0.027
	Education level		0.023
	Completion time	✓	–0.096

Abbreviations: CEFD, clinically evident Fabry disease; CVD, cardiovascular disease; ESRD, end-stage renal disease.
[a]*P* < .5. (This statistical value reflects the significance in the linear regression analysis.)

Table S2. Sensitivity Analyses

Scenario	Exclusion Criteria (n)	Pain	Moderate CEFD	Severe CEFD	ESRD	CVD	Stroke	Severe CEFD + ESRD	Severe CEFD + CVD	Severe CEFD + Stroke
Base case	NA	0.465	0.203	0.156	0.119	0.278	0.385	0.033	0.081	0.111
1	Completion time <10.5 minutes (1063)	0.459	0.177	0.133	0.091	0.265	0.375	–0.002	0.047	0.079
2	Same value for all health states (1071)	0.495	0.207	0.155	0.107	0.289	0.407	0.020	0.073	0.106
3	Pain (least severe) not best health state (652)	0.614	0.277	0.219	0.111	0.232	0.264	0.039	0.066	0.069
4	Any inconsistencies (523)	0.459	0.181	0.111	0.078	0.244	0.302	–0.087	–0.070	–0.067
5	≥2 inconsistencies (761)	0.488	0.219	0.179	0.118	0.277	0.342	–0.047	–0.019	0.000
6	≥3 inconsistencies (908)	0.487	0.218	0.195	0.118	0.277	0.356	–0.022	0.010	0.027
7	Same value for all states and ≥2 inconsistencies (657)	0.539	0.228	0.182	0.111	0.295	0.370	–0.081	–0.047	-0.026

Abbreviations: CEFD, clinically evident Fabry disease; CVD, cardiovascular disease; ESRD, end-stage renal disease; NA, not applicable.
